# Supplementary material for: Investigating the putative unforeseen link between football fervour and colorectal cancer screening in Denmark
Source: PeerJ. 2024 Sep 26;12:e18057. doi: 10.7717/peerj.18057 (PMC11439399; doi:10.7717/peerj.18057)

Supplemental Figure 1

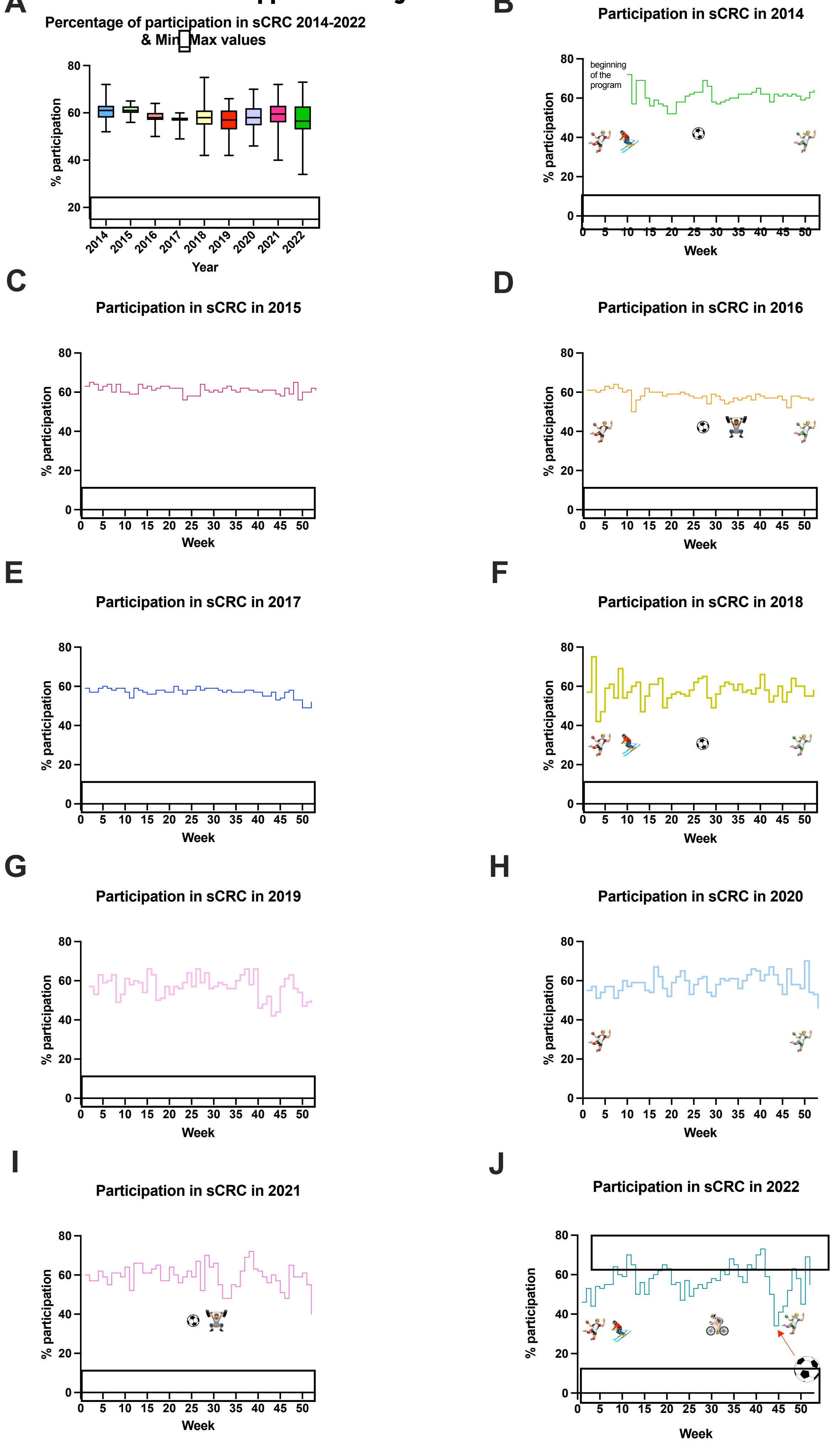

Supplement: Supplemental Information 1 — (A) General overview of the percentage of participation for all the years (2014–2022) showing the medium maximum and minimum registered values every week, the lowest point during the entire period was the first week of the FWC22, (B) Percentage of participation with the beginning of the sCRC program in Denmark in April 2014, (C) Percentage of participation for 2015, (D) Percentage of participation for 2016, (E) Percentage of participation for 2017 (data from week 51 was excluded due to the introduction of a new IT system), (F) Percentage of participation for 2018, (G) Percentage of participation for 2019, (H) Percentage of participation for 2020 (data from week 6, 12 and 13 were excluded because IAM wrongly invited a number of citizens), (I) Percentage of participation for 2021, and (J) Percentage of participation for 2022 including of the phenomenon of reduction during the 2-week when the Danish national football in the FWC22, this figure is the same as the one presented in Fig. 3, panel A in the manuscript. Sport icons used to illustrate the different sport events: Football World Cup (2014 and 2018), Tour de France (event hosted in Denmark in 2022), European Football Championship (2016 and 2021), Winter Olympics (2014, 2018, and 2022), Summer Olympics (2016, 2021) and Handball European Championships (2014, 2018, 2020 and 2022) were implemented from standard icons in software Preview version 11.0 (1042.1) and GraphPad Prism software version 10.2.2. [file peerj-12-18057-s001.pdf]
