# Supplementary material for: Investigating the putative unforeseen link between football fervour and colorectal cancer screening in Denmark
Source: PeerJ. 2024 Sep 26;12:e18057. doi: 10.7717/peerj.18057 (PMC11439399; doi:10.7717/peerj.18057)
Supplement: Supplemental Information 2 — The first column lists the date and week that the invitation is generated. At the end of the same week the invitation is sent to citizens. Column 2 lists the approximate time the citizens will receive the invitation in their mailbox. With a yearly median response time of 31 days, the week that the returned sample will be received in the lab is given in column 3. In column 4 the FWC22 matches that take place during the week that includes the median response day (column 3) are listed. [file peerj-12-18057-s002.pdf]

Table 1 | Timetable for invitations to the colorectal screening program autum 2022

Generation of invitations (week)	Invitation in citizen's mailbox late week	Expected 50% response by weeks	Relevant Football World Cup 2022 dates
24 Oct (43)	44	45 - 47	Preinaguration of World Cup 20 Nov
**31 Oct (44)**	**45**	**46 - 48**	**Opening, and Danish matches on 22 and 26 Nov**
**07 Nov (45)**	**46**	**47 - 49**	**Danish match on 30th Nov**
14 Nov (46)	47	48 - 50	Quater finals 9 and 10 Dec
21 Nov (47)	48	49 - 51	Semi finals 13 and 14 Dec
28 Nov (48)	49	50 - 52	World Cup Final 18 Dec
